# Tip-Apex Distance Is Most Important of Six Predictors of Screw Cutout After Internal Fixation of Intertrochanteric Fractures in Women

**DOI:** 10.2106/JBJS.OA.16.00022

**Published:** 2017-11-02

**Authors:** Tatsuya Fujii, Shun Nakayama, Masahiko Hara, Wataru Koizumi, Takashi Itabashi, Masahito Saito

**Affiliations:** 1 Department of Orthopedics, Narita Red Cross Hospital, Narita, Japan; 2 Department of Orthopedics, Sanno Hospital, Chiba, Japan; 3 Department of Clinical Investigation, Cloud Clinic, Takarazuka, Japan

## Abstract

**Background:**

Six risk factors for screw cutout after internal fixation of intertrochanteric fractures have been reported. The purpose of the present study was to evaluate and compare the impact of the 6 risk factors of screw cutout to clarify the most important one.

**Methods:**

We enrolled 8 consecutive patients who had screw cutout and 48 random control subjects after internal fixation of intertrochanteric fractures treated with proximal femoral nail antirotation systems at our institution. All of the patients were female. The group that had screw cutout and the control group were retrospectively evaluated and compared with respect to the OTA/AO classification, presence of a posterolateral fragment, types of reduction pattern on anteroposterior and lateral radiographic images, position of the screw, and the presence of a tip-apex distance (TAD) of ≥20 mm. The impact of each factor on screw cutout was assessed using backward stepwise multivariable logistic regression analysis with the Akaike information criterion. Risk stratification was assessed using classification and regression tree (CART) analysis.

**Results:**

Among 6 risk factors, only a TAD of ≥20 mm had a significant impact on screw cutout, with an adjusted odds ratio of 12.4 (95% confidence interval, 1.6 to 129.0; p = 0.019). CART analysis also demonstrated that a TAD of ≥20 mm was the most important risk stratification factor (p < 0.001).

**Conclusions:**

Among the 6 previously reported screw cutout-related factors, only a TAD of ≥20 mm was associated with screw cutout after internal fixation of intertrochanteric fractures with proximal femoral nail antirotation systems.

**Level of Evidence:**

Therapeutic Level III. See Instructions for Authors for a complete description of levels of evidence.

Hip fracture is defined as a femoral fracture that occurs in the proximal end of the femur and leads to serious physical and cognitive difficulties^1-4^. The number of hip fractures and hip surgeries continues to increase, as osteoporosis of the bone and poor balance ability affect the growing worldwide elderly population^5-9^. Intertrochanteric fracture is one of the most important types of hip fracture. It is generally treated with internal fixation, which, in comparison with conservative treatment, relieves pain, rapidly improves the ability to resume activities of daily living, and results in the recovery of physical function and rehabilitation into society^10-12^.

On the other hand, cutout of the screw from the femoral head has been reported as one of the most serious perioperative complications following internal fixation of intertrochanteric fractures, with an estimated prevalence of 1.9% to 3.2%^13,14^. Cutout of the screw from the femoral head is defined as “the collapse of the neck-shaft angle into varus, leading to extrusion, or so-called cutout, of the screw from the femoral head,” which needs to be addressed with reoperation under general anesthesia^15^. Thus, the identification of risk factors and the prevention of screw cutout after internal fixation of intertrochanteric fractures are important for orthopaedic surgeons. Six risk factors for screw cutout after internal fixation of intertrochanteric fractures, including 2 patient-related and 4 operation-related factors, have been reported^16,17^. However, to our knowledge, there is no comprehensive evaluation available regarding which of the 6 factors has the largest impact on the prevalence of screw cutout. The purpose of the present study was to evaluate and compare the impact of the 6 screw cutout-related factors simultaneously, in order to clarify the most important screw cutout-related factor and to discuss possible preventive strategies.

## Materials and Methods

### Study Population

**Fig. 1:**
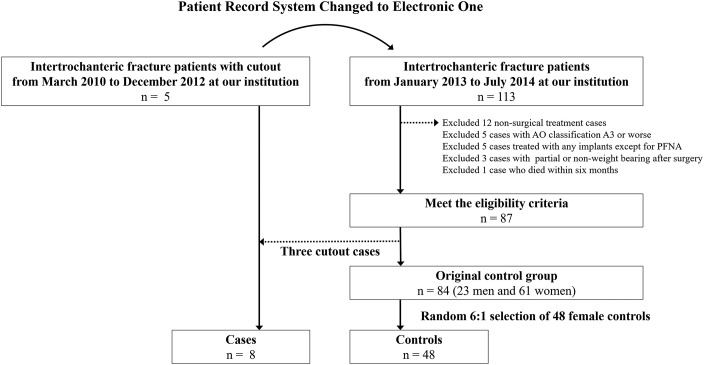
Patient selection flow chart. PFNA = proximal femoral nail antirotation.

Among the patients with intertrochanteric fractures who were admitted to Narita Red Cross Hospital and underwent surgical treatment with an intramedullary nail (proximal femoral nail antirotation [PFNA]; Synthes), 8 consecutive patients with screw cutout who were hospitalized between March 2010 and July 2014, and 48 random control subjects who were hospitalized between January 2013 and July 2014, were enrolled (Fig. 1). We selected a case-control design with a 6:1 control-to-case ratio. As all patients were women, 48 control subjects were randomly selected from among the 61 women in our original control group to minimize bias and make our study populations more homogeneous. All controls were selected from among patients after January 2013 because the hospital medical record system was changed at our institution in January 2013 (Fig. 1).

Patients were excluded if they had a fracture classified as type A3 or worse according to the OTA/AO classification system^18^, surgical treatment with any implants (except for PFNA), or any partial or non-weight-bearing condition in postoperative rehabilitation (Fig. 1). At our institution, all intertrochanteric fractures were treated with intramedullary nails, and no sliding hip screw implantations were performed in the study period. The rationale was that there was no significant difference in complication rates between the PFN and the sliding hip screw and that the PFNA is the refined version of the PFN, although the sliding hip screw was considered to be superior to the Gamma Nail (Stryker) for trochanteric fractures as stated in the Cochrane review published in 2008^10,19^.

We retrospectively collected all of the variables that are shown in the tables and figures from the patient records, and the requirement for written informed consent was waived. The cases of several patients had been previously described in case reports; hence, we obtained permission for the use of images that were later modified for inclusion in the present study^20^. The study protocol complied with the Helsinki Declaration standards and was approved by the Ethical Committee of Narita Red Cross Hospital.

### Internal Fixation and Subsequent Weight-Bearing

The patients were positioned supine on a traction table and were placed under spinal or general anesthesia. We first attempted a closed reduction approach with the use of traction along the axis of the limb with the femur placed in internal or external rotation, and with an image intensifier (C-arm) used in the preoperative planning to obtain the correct alignment and morphology of the femur with reference to the healthy side^21^. If the reduction was not appropriate, we performed a limited open reduction with an incision such as an anterior incision at the femoral neck. After confirming reduction on anteroposterior and lateral radiographs for limited open surgery, we performed internal fixation using a PFNA and 3 small incisions. During PFNA fixation, we first decided on an optimal entry point, which was at the tip of the greater trochanter, and then reamed to make it possible to prevent loss of reduction by gently introducing the nail^21,22^. The PFNA has an impaction nail component that is used for the femoral head. A helical blade measuring 10.3 mm was inserted into a nail with a 16.5-mm proximal geometry. We placed the blade in the femoral head to attain stability of the blade on anteroposterior and lateral radiographs and minimize the tip-apex distance (TAD). The position of the blade was stable when it was positioned in the central-central or the central-inferior zone of the femoral head by dividing the head into 3 zones on the anteroposterior radiograph and 3 zones on the lateral radiograph^23^. The position was plotted on the sagittal plane as seen on the postoperative radiograph to obtain better resistance against rotation of the femur after surgery^23^. Finally, we inserted a distal locking screw using the aiming arm device. Radiographic evaluations were performed preoperatively, immediately postoperatively, and once weekly for 2 months after surgery. After postoperative radiography confirmed the absence of displacement of the PFNA and the correct morphology of the fixed femur, we allowed early mobilization and full weight-bearing.

### Cutout Risk Factors

We evaluated 6 previously reported cutout risk factors that included (1) an unstable intertrochanteric fracture, which was defined using the OTA/AO classification as type A2.2 or A2.3^18^; (2) a posterolateral fracture fragment, which was defined using the Jensen classification as type III or V^24^; (3) a medial type of reduction pattern, in which the proximal fragment lay inward from the anatomical position in the postoperative anteroposterior radiograph^25^; (4) an intramedullary type of reduction pattern, in which the anterior cortex of the proximal part of the femur was located at the rear of the anterior cortex of the distal fragment in the postoperative lateral radiograph^25^; (5) an unstable position of the screw in femoral head zones other than the central-central and central-inferior zones (the position was plotted on the sagittal plane as seen on the postoperative radiograph^23^); and (6) a TAD of ≥20 mm^15,23^. The TAD is the sum of the postoperative distances from the tip of the screw to the apex of the femoral head on the anteroposterior and lateral radiographs. All calibrations were performed by referencing the PFNA blade, which was 10.3 mm in diameter. The first 2 factors were considered to be preoperative and the others were considered to be operative (Table I).

**TABLE I:** Patient Characteristics

Parameters	Cases (N = 8)	Controls (N = 48)	P Value
Age* *(yr)*	83 (77-88)	85 (80-90)	0.399
PFNA length *(no. [%])*			0.743
170 mm	5 (62.5)	26 (54.2)	
200 mm	3 (37.5)	19 (39.6)	
240 mm	0 (0.0)	3 (6.3)	
Open reduction† *(no. [%])*	2 (25.0)	9 (19.1)	0.702
Risk factors for cutout *(no. [%])*			
Preoperative			
Unstable intertrochanteric fracture	4 (50.0)	15 (31.3)	0.300
OTA/AO classification A2.2	3 (37.5)	11 (22.9)	
OTA/AO classification A2.3	1 (12.5)	4 (8.3)	
Posterolateral fragment	4 (50.0)	19 (39.6)	0.579
Operative			
Medial type of reduction	5 (62.5)	5 (10.4)	<0.001
Intramedullary type of reduction	4 (50.0)	17 (35.4)	0.430
Unstable position of the screw	5 (62.5)	5 (10.4)	<0.001
TAD of ≥20 mm	5 (62.5)	4 (8.3)	<0.001
Weeks from internal fixation to cutout*	7 (4-13)	–	–

* The values are given as the median and the 25th to 75th percentiles.

† Data are missing on one patient who had open reduction in the control group.

### Statistical Analysis

We set the postoperative cutout of the screw from the femoral head within 6 months after the operation as the primary end point. A cutout was defined as the collapse of the neck-shaft angle into varus, leading to extrusion of the screw from the femoral head. Continuous variables were summarized using medians and interquartile ranges (quartiles 1 to 3), and categorical variables were summarized by means of counts and percentages. We used the Wilcoxon rank-sum test for comparisons of continuous variables and the chi-square test for comparisons of categorical variables between cutout case and control groups. The impact of each risk factor on cutout was assessed using univariable and multivariable logistic regression analysis by estimating the odds ratio (OR) and its 95% confidence interval (CI). Covariates included all 6 previously reported cutout risks and age (Table II). For the multivariable model selection, we employed a backward stepwise method with the Akaike information criterion to improve model fit.

**TABLE II:** Impact of Each Risk Factor on the Frequency of Cutout Using Univariable and Multivariable Logistic Regression Analysis

	Univariable			Backward AIC Model*		
Parameters	OR	95% CI	P Value	Adjusted OR	95% CI	P Value
Preoperative						
Unstable intertrochanteric fracture	2.2	0.5-10.5	0.307	–	–	–
Posterolateral fragment	1.5	0.3-7.2	0.581	–	–	–
Operative						
Medial type of reduction	14.3	2.7-90.4	0.002	7.0	0.9-68.1	0.066
Intramedullary type of reduction	1.8	0.4-8.6	0.435	–	–	–
Unstable position of the screw	14.3	2.7-90.4	0.002	7.0	0.9-68.1	0.066
TAD of ≥20 mm	18.3	3.4-123.5	0.001	12.4	1.6-129.0	0.019

* AIC = Akaike information criterion.

**Fig. 2:**
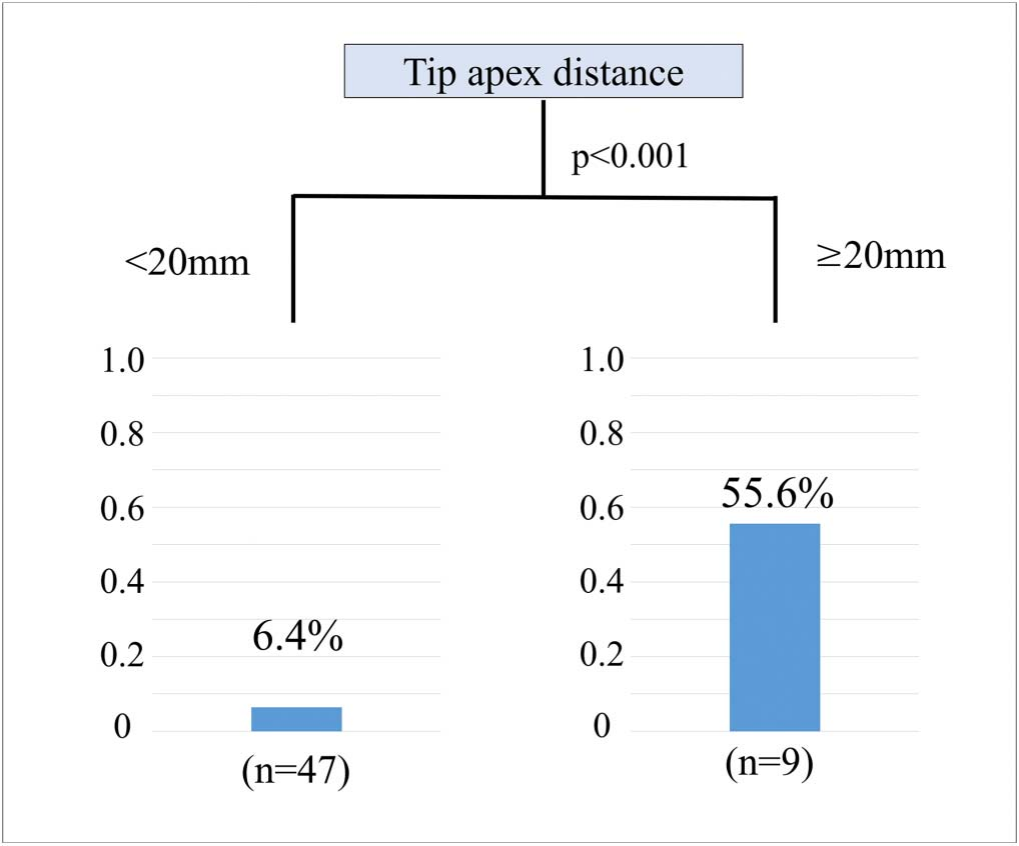
Results of CART analysis and frequency of cutout for each category. The CART analysis was performed by including age and the 6 risk factors of cutout as possible indices. The Y axis indicates the percentage of cutout among patients in each category. In the category involving a TAD of <20 mm, 6.4% (3) of 47 patients had cutout. In the category involving a TAD of ≥20 mm, 55.6% (5) of 9 patients had cutout.

In addition, classification and regression tree (CART) analysis was also used to evaluate the most important determinants of the primary end point. This analysis included age and the 6 risk factors of screw cutout as possible indices (Fig. 2). CART is a statistical method that recursively partitions the data space in order to find heterogeneous subgroups, and it is well suited for the development of clinical decision rules and risk stratification^26,27^. All statistical analyses were performed using R software packages (version 3.2.1; R Development Core Team).

## Results

In this retrospective case-control observational study, we enrolled 8 patients who had screw cutout and 48 control subjects. Patient characteristics and the frequencies of the 6 previously reported screw cutout risk factors are summarized in Table I. The median age of the patients with screw cutout and the control subjects was 83 and 85 years, respectively. All of the screw cutout cases occurred in female patients, and thus the control group was limited to women (Fig. 1). With regard to screw cutout risk factors, there were no significant differences in the frequency of preoperative risks, such as an unstable intertrochanteric fracture and a posterolateral fragment, between the case and control groups. The frequencies of 3 of 4 operative risk factors, namely, medial type of reduction, unstable position of the screw, and a TAD of ≥20 mm, were significantly higher in patients who had screw cutout than in the control group. The screw cutouts occurred after a median of 7 weeks (range, 4 to 13 weeks) following the operation.

**Fig. 3:**
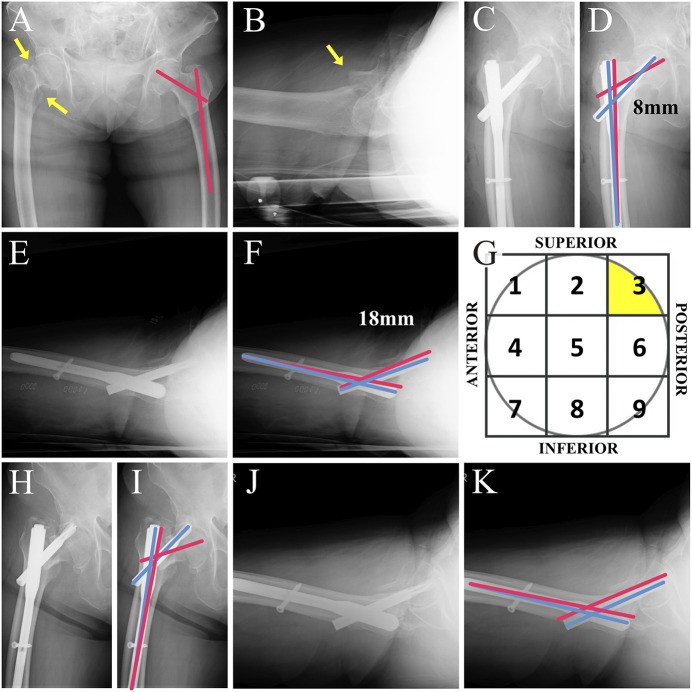
An 80-year-old woman with an intertrochanteric fracture of the right hip who had a cutout at 6 weeks after implantation with a PFNA. (Reproduced, with modification, from the Journal of Typical Medical Images and Videos. Video 2016: Case 178. http://the jtmiv.com. Reproduced with permission.) Anteroposterior (**Figs. 3-A, 3-C, 3-D, 3-H, and 3-I**) and lateral (**Figs. 3-B, 3-E, 3-F, 3-J, and 3-K**) radiographs made at the time of admission (**Figs. 3-A and 3-B**), immediately after PFNA implantation (**Figs. 3-C through 3-F**), and 6 weeks after the implantation (**Figs. 3-H through 3-K**). Ideal and practical axes for the lag screw and the nail are indicated with pink and blue lines, respectively. The operation was performed using a 130° nail; however, on retrospective review of this case, we believe a better template would have been a 125° nail. The position of the screw on the femoral head was placed in the superior-posterior zone (zone 3; **Fig. 3-G**). The TAD was calculated to be 26 mm (**Figs. 3-D and 3-F**), and the malreduction remained on the lateral radiograph (**Figs. 3-J and 3-K**). Unfortunately, the patient had a cutout at 6 weeks after the implantation (**Figs. 3-H through 3-K**).

The impact of each risk factor on the frequency of cutout was determined using univariable and multivariable logistic regression analysis (Table II). Among the 6 previously reported risk factors, only a TAD of ≥20 mm had a significant impact on cutout, with an adjusted OR of 12.4 (95% CI, 1.6 to 129.0; p = 0.019). Furthermore, CART also demonstrated that a TAD of ≥20 mm was the most important risk stratification factor (Fig. 2). We present a representative case of cutout in Figure 3.

## Discussion

In this retrospective case-control observational study, we evaluated and compared the impact of 6 previously reported risk factors on the frequency of screw cutout using multivariable logistic regression model by enrolling 8 patients who had screw cutout and 48 controls. We demonstrated that only a TAD of ≥20 mm had a significant impact on the frequency of screw cutout after internal fixation of intertrochanteric fractures. To our knowledge, this report is the first to simultaneously compare 6 screw cutout risk factors after internal fixation of intertrochanteric fractures.

### Preoperative Risks

Historically, unstable intertrochanteric fractures and posterolateral fracture fragments have been reported as preoperative risks of screw cutout. These are known as *fracture patterns*, which were defined by the OTA/AO classification or Evans classification system^23,24,28^. In our study, however, there were no significant differences in the frequency of unstable intertrochanteric fractures and posterolateral fragments between the group that had screw cutout and the control group. In addition, these 2 preoperative factors had no significant impact on the frequency of screw cutout on the basis of the results of logistic regression analysis, whereas these preoperative risks of screw cutout were reported to be important in previous studies (Table II). Considering the designs and results of previous studies, we speculated that this discrepancy between the results from previous studies and our study can be explained by the following possible mechanism. These preoperative risks could result in increased TAD if the ensuing difficulty in performing the reduction results in a remaining varus deformity. This is intuitively understandable for orthopaedic surgeons if there is difficulty in internal fixation of complex fractures with a higher OTA/AO classification or in the presence of a posterolateral fragment^16,23-25,29^. Since previous studies suggesting unstable intertrochanteric fractures and posterolateral fragments as risks of cutout did not use other operative risks, such as TAD, they could not exclude the confounding between preoperative and operative risks^29-32^. Thus, it is possible that the results from previous reports might be influenced by a potential confounding between the difficulty of the operation and operative risks such as a TAD of ≥20 mm as shown in the present study^28,30^.

### Operative Risks

Medial or intramedullary types of reduction patterns, an unstable position of the screw in the femoral head, and a TAD of ≥20 mm have been reported as operative cutout risks^15,23,25^. Among these 4 operative risk factors, only the intramedullary type of reduction did not show significant differences in the frequencies between the case and control groups. In addition, the medial type of reduction and unstable position of the screw did not have a significant impact on cutout in a multivariable model. As mentioned above, we speculated that these 3 operative risks could also lead to an increasing TAD, because the axis of the proximal fragment with an inserted lag screw was out of alignment with that of the distal fragment of the intertrochanteric fracture. The presence of mismatched axes would make it difficult for orthopaedic surgeons to achieve both a stable position of the screw and a TAD of <20 mm, resulting in positive results for medial and intramedullary types of reductions or an unstable position of the screw, as seen in previous reports^25^. Other possible reasons for the discrepancy between our results and previous reports include the relatively small number of patients and low statistical power used to detect the significant impact of multiple risk factors. However, since a TAD of ≥20 mm was identified as a major cutout risk in the multivariable logistic regression model and CART method, and since there was plenty of evidence regarding the TAD risk, it is clear that a TAD of ≥20 mm is the most important screw cutout risk factor after internal fixation of intertrochanteric fractures^15,16,23,31,32^.

### Clinical Implications

On the basis of our findings, we believe that it is important for orthopaedic surgeons to consider the TAD so as to avoid a cutout during internal fixation of an intertrochanteric fracture. The most important and practical way to achieve this condition is by the nail insertion into the femoral shaft, because the position of the nail determines the position of the screw and the resultant TAD. If we insert the nail into the femoral shaft without imaging for an ideal position of the screw in the femoral head, then the nail position may force surgeons to place the screw in an inappropriate position, which can then lead to a wide TAD. With this point of view, sliding hip screws may have an advantage, compared with the PFNA, in that screw cutout could be avoided because the screw is placed first and with the best control, although this continues to be a controversial topic in this field^12,17^. Finally, as a reminder, surgeons should not seek to minimize the TAD further when it is <20 mm during insertion of the blade. These speculations should be evaluated in a randomized controlled trial to reach definite conclusions.

### Study Limitations

There are several limitations that warrant mentioning. First, there might be a selection bias because we only enrolled patients who underwent PFNA implantation and because we excluded several patients, such as those with a partial or non-weight-bearing condition, even though cutout did not happen in these patients. Second, there were no male patients, which may be because the frequency of intertrochanteric fracture is higher in females than in males^16,17^. Third, it is possible that low occurrence rates precluded any significance in the correlation between the fracture pattern and cutout. This is unavoidable, even in a large clinical study, because the frequency of cutout is very small^17^. The low frequency of cutout cases also led to very large 95% CIs as shown in Table II. However, the important point is that the impact of the TAD operative risk might exceed those of fracture patterns because the TAD risk showed significance even after multivariable adjustment or in a CART analysis. Fourth, only very limited data on patient characteristics were available, and bone quality might have been a characteristic of interest. Lastly, the timing of the control group differs from that of the case group, which could have introduced bias (Fig. 1). Thus, our results need to be interpreted in light of these study limitations.

### Overview

Among 6 previously reported cutout-related factors, only a TAD of ≥20 mm was associated with screw cutout after internal fixation of intertrochanteric fractures.

## Disclosure of Potential Conflicts of Interest

DISCLOSURE OF POTENTIAL CONFLICTS OF INTEREST
